# Calcium and IP3 dynamics in cardiac myocytes: experimental and computational perspectives and approaches

**DOI:** 10.3389/fphar.2014.00035

**Published:** 2014-03-06

**Authors:** Felix Hohendanner, Andrew D. McCulloch, Lothar A. Blatter, Anushka P. Michailova

**Affiliations:** ^1^Department of Molecular Biophysics and Physiology, Rush University Medical CenterChicago, IL, USA; ^2^Department of Bioengineering, University of California San DiegoLa Jolla, CA, USA

**Keywords:** Ca^2+^, IP_3_, excitation-contraction coupling, excitation-transcription coupling, cardiomyocyte

## Abstract

Calcium plays a crucial role in excitation-contraction coupling (ECC), but it is also a pivotal second messenger activating Ca^2+^-dependent transcription factors in a process termed excitation-transcription coupling (ETC). Evidence accumulated over the past decade indicates a pivotal role of inositol 1,4,5-trisphosphate receptor (IP_3_R)-mediated Ca^2+^ release in the regulation of cytosolic and nuclear Ca^2+^ signals. IP_3_ is generated by stimulation of plasma membrane receptors that couple to phospholipase C (PLC), liberating IP_3_ from phosphatidylinositol 4,5-bisphosphate (PIP_2_). An intriguing aspect of IP_3_ signaling is the presence of the entire PIP_2_-PLC-IP_3_ signaling cascade as well as the presence of IP_3_Rs at the inner and outer membranes of the nuclear envelope (NE) which functions as a Ca^2+^ store. The observation that the nucleus is surrounded by its own putative Ca^2+^ store raises the possibility that nuclear IP_3_-dependent Ca^2+^ release plays a critical role in ETC. This provides a potential mechanism of regulation that acts locally and autonomously from the global cytosolic Ca^2+^ signal underlying ECC. Moreover, there is evidence that: (i) the sarcoplasmic reticulum (SR) and NE are a single contiguous Ca^2+^ store; (ii) the nuclear pore complex is the major gateway for Ca^2+^ and macromolecules to pass between the cytosol and the nucleoplasm; (iii) the inner membrane of the NE hosts key Ca^2+^ handling proteins including the Na^+^/Ca^2+^ exchanger (NCX)/GM1 complex, ryanodine receptors (RyRs), nicotinic acid adenine dinucleotide phosphate receptors (NAADPRs), Na^+^/K^+^ ATPase, and Na^+^/H^+^ exchanger. Thus, it appears that the nucleus represents a Ca^2+^ signaling domain equipped with its own ion channels and transporters that allow for complex local Ca^2+^ signals. Many experimental and modeling approaches have been used for the study of intracellular Ca^2+^ signaling but the key to the understanding of the dual role of Ca^2+^ mediating ECC and ECT lays in quantitative differences of local [Ca^2+^] in the nuclear and cytosolic compartment. In this review, we discuss the state of knowledge regarding the origin and the physiological implications of nuclear Ca^2+^ transients in different cardiac cell types (adult atrial and ventricular myocytes) as well as experimental and mathematical approaches to study Ca^2+^ and IP_3_ signaling in the cytosol and nucleus. In particular, we focus on the concept that highly localized Ca^2+^ signals are required to translocate and activate Ca^2+^-dependent transcription factors (e.g., nuclear factor of activated T-cells, NFAT; histone deacetylase, HDAC) through phosphorylation/dephosphorylation processes.

Calcium is a pivotal signaling molecule and its intracellular concentration ([Ca^2+^]_i_) is precisely regulated in different subcellular domains. The modulation of [Ca^2+^]is a crucial factor for a variety of physiological functions of living cells. In cardiac myocytes, including ventricular and atrial cells, Ca^2+^ release through channels located in the sarcoplasmic reticulum (SR) membrane and termed ryanodine receptors (RyRs), is a key event linking membrane depolarization and mechanical activity during excitation-contraction coupling (ECC) (Bers, 2001). The amount of Ca^2+^ release with each heart beat and by that the force of contraction is also modulated by hormonal action, e.g., by Endothelin I and Angiotensin II (Proven et al., 2006). These two hormones stimulate plasma membrane receptors (G protein coupled receptors, GPCRs) that couple to phospholipase C (PLC), liberating IP_3_ from phosphatidylinositol 4,5-bisphosphate (PIP_2_). IP_3_ freely diffuses within the cytoplasm to bind to a second type of SR Ca^2+^ release channels, the inositol 1,4,5-trisphosphate receptor (IP_3_R) (Roderick and Bootman, 2003; Kockskämper et al., 2008; Berridge, 2009). IP_3_Rs, albeit at a much smaller density compared to ryanodine receptors (RyR:IP_3_R ~100:1), are expressed in the SR membrane and nuclear envelope (NE) (Bootman et al., 2009). The activation of IP_3_Rs upon binding of IP_3_ can modulate ECC by sensitizing nearby RyRs leading to positive inotropic but also pro-arrhythmic effects (Petersen et al., 1994; Vogelsand et al., 1994; Zima and Blatter, 2004; Harzheim et al., 2009). Experimental evidence accumulated over the past decade also indicates an important role of IP_3_R-mediated Ca^2+^ release in excitation-transcription coupling (ETC) and pro-hypertrophic signaling (Arantes et al., 2012). The entire PIP_2_-PLC-IP_3_ cascade, including GPCRs and IP_3_Rs, can be found in the NE (Bkaily et al., 2011; Vaniotis et al., 2011; Tadevosyan et al., 2012). The presence of nuclear GPCRs in combination with highly localized nuclear IP_3_R-mediated Ca^2+^ release and Ca^2+^ removal might provide for a putative distinct signaling domain that regulates nuclear Ca^2+^ dynamics (e.g., for autocrine signaling), whereas the cytosolic Ca^2+^ is regulated separately via sarcolemmal GPCR signaling and IP_3_R-mediated SR Ca^2+^ release in conjunction with Ca^2+^ release and removal by the set of proteins involved in ECC (e.g., RyR, SERCA, troponin C). Sarcolemmal GPCRs allow for paracrine signaling and positive inotropic effects mediated by hormonal stimulation (e.g., with Angiotensin II or Endothelin I), (Kockskämper et al., 2008; Bootman et al., 2009). A comprehensive understanding of the mechanisms regulating nuclear IP_3_ and Ca^2+^ signals and the impact of alterations of cytosolic Ca^2+^ and IP_3_ signals on nuclear functions requires well-characterized experimental approaches, but also whole-cell system mathematical models. In this review, we discuss quantitative aspects of IP_3_-dependent Ca^2+^ homeostasis in adult ventricular and atrial myocytes. In particular, we focus on novel modeling and experimental approaches to support the concept that IP_3_R-mediated Ca^2+^-release and the Ca^2+^ removal machinery in the SR and NE allow for highly localized and independent cellular signaling.

## Excitation-contraction coupling in ventricular and atrial myocytes and the role of IP_3_

**Figure 1:**
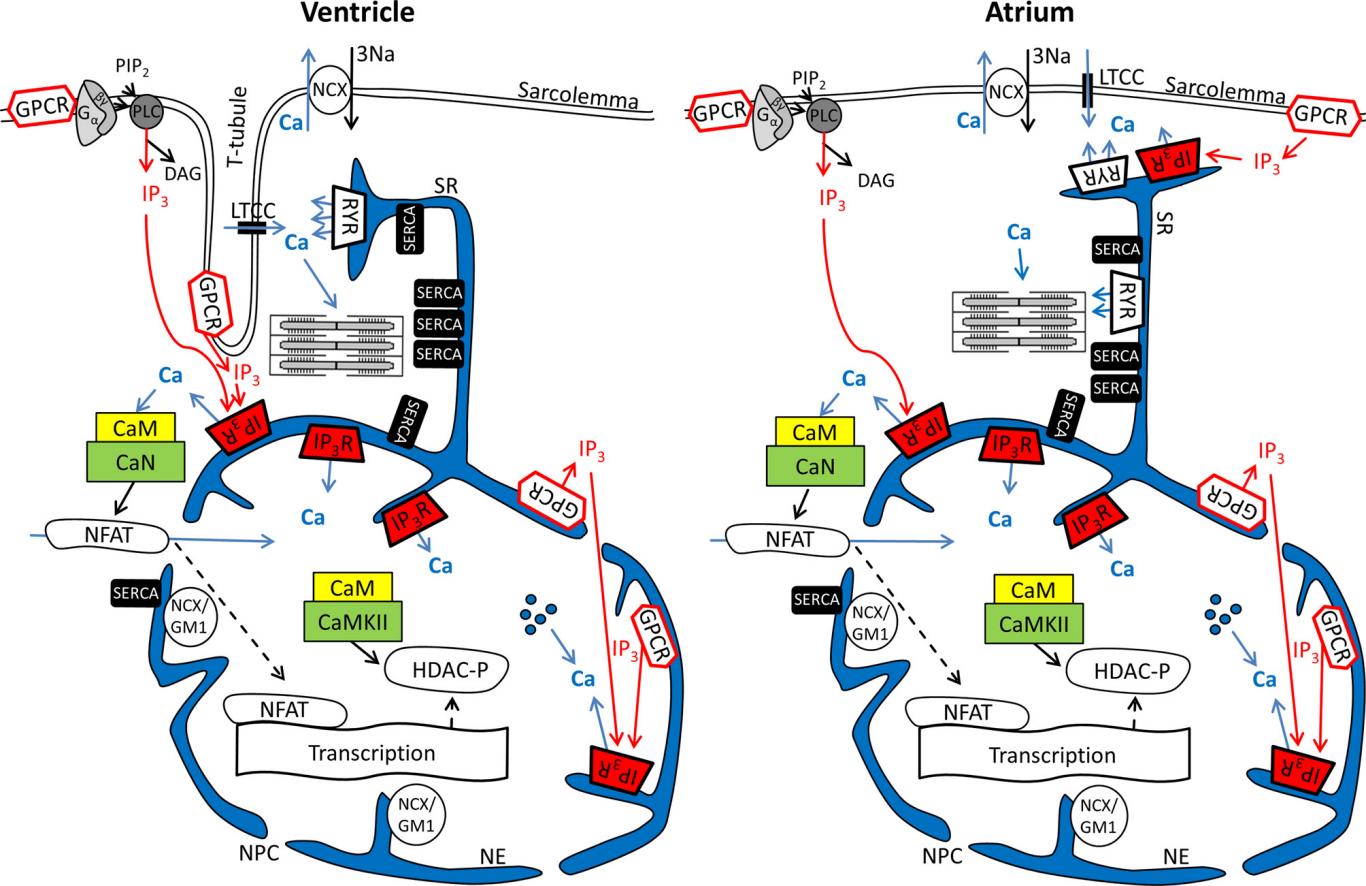
**Ca^2+^ and IP_3_ and their involvement in cardiac excitation-contraction and excitation-transcription coupling in ventricular and atrial myocytes.** Schematics depict parts of the sarcolemmal plasma membrane as well sarcoplasmic reticulum and nuclear envelope as a contiguous Ca^2+^ store. Abbreviations: GPCR, G protein-coupled receptor; PIP_2_, phosphatidylinositol 4,5-bisphosphate; PLC, phospholipase C; LTCC, L-type Ca^2+^ channel; NCX, Na^+^/Ca^2+^ exchanger; RyR, ryanodine receptor; IP_3_R, IP_3_ receptor; SERCA, SR Ca^2+^ ATPase; NCX/GM1, NCX ganglioside complex; CaM, Calmodulin; CaN, Calcineurin; CaMKII, Ca-Calmodulin dependent kinase; NFAT, nuclear factor of activated t-cells; DAG, Diacylglycerol; HDAC, histone deacetylase; NPC, nuclear pore complex; 
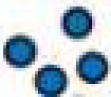
 Ca^2+^ released from intra-nuclear pools.

**Figure 2:**
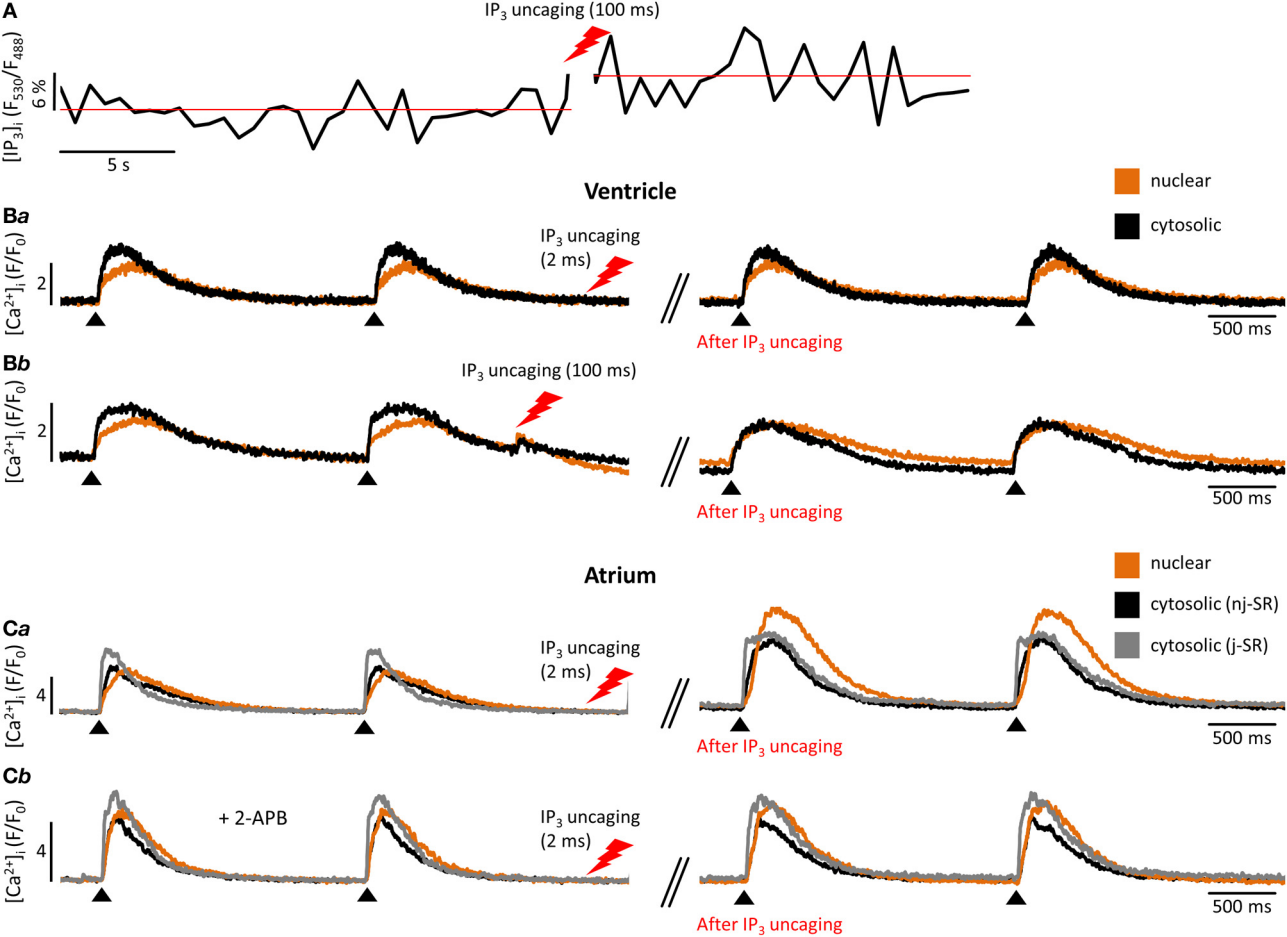
**Experimental measurement of cytosolic and nuclear Ca^2+^ and IP_3_. (A)** Cardiomyocyte loaded with the caged IP_3_ compound cag-IP_3_ PM and expressing cytosolic FIRE-1. Whole cell [IP_3_] signal shown as changes in FIRE-1 FRET signal (expressed as F_530_/F_488_) after global IP_3_ uncaging by exposure to 405 nm laser illumination for 100 ms. **(B*a,b*)** Effects of global IP_3_ uncaging (2 ms or 100 ms illumination) in field-stimulated ventricular myocytes on global nuclear and cytosolic Ca^2+^ transients. **(C*a*)** The effect of global IP_3_ uncaging (2 ms) on global nuclear, subsarcolemmal (j-SR) and central (nj-SR) Ca^2+^ transients in field stimulated atrial myocyte. **(C*b*)** The effect of global IP_3_ uncaging (2 ms) on global nuclear, j-SR and central Ca^2+^ transients in field stimulated atrial myocyte pre-incubated with the IP_3_R blocker 2-APB. Pacing frequency 0.5 Hz. Black arrowheads indicate application of electrical stimuli.

In cardiomyocytes, ECC describes the process of action potential (AP) triggered Ca^2+^-induced Ca^2+^ release (CICR) providing sufficient Ca^2+^ for the activation of the proteins regulating muscle contraction and to induce active muscle force (Bers, 2001). Membrane depolarization during an AP allows Ca^2+^ influx through voltage-dependent L-type Ca^2+^ channels (LTCC) which triggers CICR and thereby amplifies the cytosolic Ca^2+^ signal to levels required for the activation of the contractile proteins. An important feature of all *ventricular myocytes*, setting them apart from most atrial cells, is the presence of plasma membrane invaginations throughout the cytosol (transverse or t-tubules), putting LTCC in close vicinity to RyRs (Figure 1). The SR containing RyRs that oppose LTCC is called junctional SR (jSR). The jSR is crucial for the spatiotemporal homogeneity of Ca^2+^ release leading to largely uniform cytosolic Ca^2+^ transients ([Ca^2+^]_i_) during a *ventricular cell* twitch (Figure 2), (Franzini-Armstrong et al., 1999; Heinzel et al., 2002; Louch et al., 2004; Crossman et al., 2011; Hake et al., 2012; Signore et al., 2013). Unlike in ventricular cells, the t-tubular system in *atrial myocytes* is either absent (Figure 1) (Hüser et al., 1996; Kockskämper et al., 2001) or poorly developed (Kirk et al., 2003). However more recent work in sheep and human has provided evidence that atrial cells from larger animals tend to have a higher density of t-tubules (Dibb et al., 2009; Richards et al., 2011), and even in rodent atrial cells an irregular internal transverse-axial tubular system has been identified that affects kinetics of SR Ca^2+^ release (Kirk et al., 2003).The absence or paucity of t-tubules in atrial cells leads to great differences in the shape and kinetics of local Ca^2+^ transients and gradients in subcellular regions where Ca^2+^ is provided by release from jSR and non-junctional SR (njSR) (Figure 2). Subsarcolemmal Ca^2+^ transients rise faster, have a higher Ca^2+^ peak and are initiated by Ca^2+^ currents through LTCCs, followed by RyR-mediated Ca^2+^ release from the jSR. These local jSR Ca^2+^ transients resemble Ca^2+^ release in ventricular cells. Central cytosolic Ca^2+^ transients, however, have a slower rise time and a lower peak, and result from CICR that propagates in a Ca^2+^ wave-like fashion from the periphery to the center of the cell. (Blatter et al., 2003; Maxwell and Blatter, 2012). Furthermore, the specific topological organization of the plasma membrane in atrial myocytes leads not only to different spatial [Ca^2+^]_i_ distribution as compared to the ventricle, it also affects nuclear Ca^2+^ transients by further delaying their onset due to the wave-like propagation of Ca^2+^ toward the nucleus (Figure 2). Interestingly, for both atrial and ventricular cells, a role of cytosolic IP_3_ ([IP_3_]_i_) has been reported for the modulation of cytosolic Ca^2+^ transients in a variety of animal models (Zima and Blatter, 2004; Proven et al., 2006; Domeier et al., 2008; Harzheim et al., 2009; Kim et al., 2010). IP_3_R channel activity, with type-2 IP_3_Rs as the most prevalent isoform in cardiac myocytes, depends on [IP_3_]_i_ and [Ca^2+^]_i_ (Michell et al., 1981; Domeier et al., 2008; Kockskämper et al., 2008). There is evidence that atrial myocytes express functional IP_3_Rs at higher densities than ventricular myocytes (Figure 1; in ventricular cell the IP_3_Rs are not shown in the junctional space due their relatively low density) (Mackenzie et al., 2004; Zima and Blatter, 2004). As shown in Figure 2, the acute increase in cytosolic IP_3_, induced by photolytic release of IP_3_ from a caged IP_3_ compound, increases cytosolic Ca^2+^ transient peak amplitudes during field stimulation in atrial cells in contrast to ventricular cells. In ventricular cells only increased expression levels of IP_3_R, as it occurs in cardiac hypertrophy, could experimentally be tied to enhanced cytosolic SR Ca^2+^ release (Harzheim et al., 2009). The neurohumoral stimulation with Endothelin I or Angiotensin II, however, has been shown to have similar positive inotropic effects in both ventricular and atrial cells, indicating a role of IP_3_-mediated Ca^2+^ release in the enhancement of cytosolic Ca^2+^ release (Zima and Blatter, 2004).

## Excitation-transcription coupling in ventricular and atrial myocytes and the role of IP_3_

Nuclear Ca^2+^ signals however are different with regards to kinetics during action potential induced Ca^2+^ transients. This can largely be attributed to the fact that the nucleus is surrounded by the nuclear envelope (Kockskämper et al., 2008; Alonso and García-Sancho, 2011), consisting of the outer and inner nuclear membranes and the space between them that is contiguous with the SR (Wu et al., 2006; Shkryl et al., 2012). The nuclear membranes fuse at many locations to form pores (diameter ~100, length ~50 nm) that harbor the nuclear pore complexes (NPCs). The NPCs are the major gateway for ions (including Ca^2+^) to diffuse along the gradient between the cytosol and nucleoplasm. It has been proposed that NPCs can act as diffusion filter and introduce a kinetic delay in the equilibration of nucleoplasmic Ca^2+^ concentration ([Ca^2+^]_nuc_) and [Ca^2+^]_i_ (Bootman et al., 2009). The extent of the kinetic delay might be subject to modulation. Although NPCs do not close, their conductance can change in response to factors such as Ca^2+^ and ATP. The density of NPCs can vary from 1 to 5 NPCs per μm^2^, depending on the cell type (Wang and Clapham, 1999). A greater expression of NPCs would allow for a more rapid equilibration of [Ca^2+^]_i_ and [Ca^2+^]_nuc_. Recent data from Alonso and García-Sancho (2011) also suggest a role for NE invaginations (nucleoplasmic reticulum) and intra-nuclear Ca^2+^ pools for the regulation of nuclear Ca^2+^ (Figure 1). More evidence that nuclear Ca^2+^ dynamics are not just a function of cytosolic Ca^2+^ transients can be found in structural and functional differences of NE Ca^2+^ handling proteins as compared to the SR. Even though the NE is an extension of the SR (Wu et al., 2006; Shkryl et al., 2012) SERCA presumably is not expressed at the inner NE membrane (Malviya and Klein, 2006; Bootman et al., 2009). Nonetheless, other putative Ca^2+^ handling and ion transporting proteins have been suggested to be present in the NE, including a splice variant of the type-1 Na^+^/Ca^2+^ exchanger associated with ganglioside (NCX/GM1 complex, typical for non-excitable cells), RyRs, NAADPR (nicotinic acid adenine dinucleotide phosphate receptor), Na^+^/K^+^ ATPase and Na^+^/H^+^ exchanger (Gerasimenko et al., 2003; Irvine, 2003; Bkaily et al., 2006; Ledeen and Wu, 2007; Zima et al., 2007; Guatimosim et al., 2008; Wu et al., 2009).

Even more important seems the preferential expression of IP_3_Rs in the NE (Bare et al., 2005). Using Fluo-5N Zima et al. observed a depletion of the nuclear envelope upon experimental stimulation of IP_3_Rs with IP_3_ in isolated nuclei (Zima et al., 2007) that was paralleled by an increase of [Ca^2+^]_nuc_. Wu and colleagues obtained similar results with Fluo-5N on IP_3_ dependent NE Ca^2+^ depletion in permeabilized cells (Wu et al., 2006). The importance of IP_3_ for the regulation of [Ca^2+^]_nuc_ is underscored by the results shown in Figure 2: Following cell-wide IP_3_ uncaging, nuclear Ca^2+^ transients are consistently and preferentially altered in atrial and ventricular cells. However since IP_3_ is buffered (i.e., by IP_3_Rs) and degraded over time (Woodcock and Matkovich, 2005), the subcellular localization of IP_3_Rs and the site of IP_3_ generation (i.e., GPCR) are important to generate highly localized Ca^2+^ signals to control Ca^2+^-dependent transcription (Bers, 2013; Ibarra et al., 2013). The traditional view on the positioning of GPCRs in cardiac myocytes sees their main site of expression in the sarcolemmal and nuclear membrane (Figure 1). Only recently, work from Ibarra et al. (2013) suggested a third type of localization for GPCRs in t-tubules close to the nuclear envelope (Figure 1, ventricular cell). The positioning of IP_3_ production and IP_3_Rs is important since differences in the kinetics of local [Ca^2+^] can lead to altered activation of transcription factors. A pronounced local elevation of [Ca^2+^] for instance, can activate calmodulin dependent-protein kinase II (CaMKII) and promote histone deacetylases (HDAC) phosphorylation (Wu et al., 2006), whereas a sustained smaller [Ca^2+^] elevation increases nuclear factor of activated T-cells (NFAT) dephosphorylation via the Ca^2+^ sensitive phosphatase calcineurin (CaN). This ultimately leads to the activation of different sets of transcription factors, e.g., myocyte enhancer factor 2 (MEF2) for HDAC and GATA for NFAT (Molkentin et al., 1998). The separate set of Ca^2+^ release and removal proteins in the NE, with IP_3_Rs as the most prominent example, as well as the specific expression of GPCRs in the sarcolemmal and nuclear membranes might be key to understanding the conundrum of Ca^2+^ being a modulator of contraction and transcription at the same time (Bootman et al., 2009). Mathematical modeling of nuclear and cytosolic Ca^2+^ homeostasis, accounting for different expression levels of sarcolemmal, cytosolic and nuclear Ca^2+^ handling proteins, paralleled by experimental approaches might provide a better understanding of functional differences of nuclear and cytosolic Ca^2+^.

## Experimental tools for measuring cytosolic and nuclear Ca^2+^ and IP_3_ signals

Confocal laser microscopy, multiphoton imaging and conventional microscopy provide the basis for visualization of whole cell and subcellular ion concentration distributions, and the development of chemical fluorescent Ca^2+^ indicators (Grynkiewicz et al., 1985) made imaging of Ca^2+^ movements inside living cells feasible. Nowadays a variety of ratiometric and non-ratiometric Ca^2+^ indicators, with Indo-1 and Fluo-4 among the most prominent examples, are being used. In principle, upon excitation, these indicators emit light at particular wave lengths and the emitted fluorescence intensity or the emission spectrum is changed in a Ca^2+^ bound state (Takahashi et al., 1999). The dissociation constant (K_*d*_) as a measure of Ca^2+^ binding affinity is crucial for the selection of the appropriate Ca^2+^ dye for a particular cellular compartment of interest. Low affinity, high K_*d*_ dyes (like Fluo-5N) are used for the visualization of changes in SR [Ca^2+^] or nuclear envelope [Ca^2+^], whereas, e.g., Fluo-4 (K_*d*_ of 345 nM) is one of the preferred dyes for imaging of changes in cytosolic free [Ca^2+^], which varies roughly between 100 nM and values at times exceeding 1 μM during ECC. Since the nucleoplasm and the cytoplasm are interconnected compartments with similar global [Ca^2+^] characteristics, dyes suitable to show changes in [Ca^2+^]_i_ can be used for the detection of changes in [Ca^2+^]_nuc_ as well. Using Ca^2+^ sensitive dyes, Zima and Blatter (2004) were able to visualize cytosolic IP_3_R-mediated Ca^2+^ release events (Ca^2+^ puffs) and show a positive inotropic effect of neurohumoral stimulation with Endothelin-1 in cardiac myocytes. As mentioned above, the same group was also able to show changes of local nuclear envelope [Ca^2+^] in isolated nuclei upon stimulation with IP_3_, using Fluo-5N (Zima et al., 2007).

A variety of pharmacologic interventions can be used to influence the IP_3_-dependent signaling cascade. Tools for stimulation of the neurohumoral GPCR pathway in cardiomyocytes include for example Angiotensin II and Endothelin-1. PLC-inhibitors like U73122 and IP_3_R blockers like 2-Aminoethoxydiphenyl borate (2-APB) or heparin are widely used IP_3_R blockers to study the GPCR/PLC/IP_3_ pathway. More recent molecular techniques and the generation of transgenic animals complement these tools. Noteworthy are the generation of IP_3_R knock-out and IP_3_R overexpressing mice as well as the development of IP_3_-sponges that allows the cellular overexpression of IP_3_ buffering proteins. The generation of IP_3_R overexpressing mice combined with the adenoviral expression of an IP_3_ sponge provided novel insights into the importance of this pathway in cardiac physiology and pathophysiology. Ca^2+^ transients in IP_3_R overexpressing mice were increased and showed a higher potential for arrhythmias after Endothelin-1 treatment. These effects were abrogated after expression of the IP_3_ sponge (Nakayama et al., 2010). Insensitivity toward GPCR stimulation and IP_3_R-mediated pro-arrhythmic effects were confirmed in IP_3_R knock-out mice (Li et al., 2005).

An approach to directly visualize cellular [IP_3_] would allow for a more complete picture of cell physiology. Only recently Remus et al. (2006) developed biosensors termed FIRE to dynamically study [IP_3_] in living cells. Briefly, FIRE is incorporated into an adenoviral vector, expressed in target cells, and utilizes fluorescence resonance energy transfer (FRET) between cyan and yellow fluorescent protein (CFP and YFP) upon binding of IP_3_. For that purpose FIRE contains a fusion protein of CFP, YFP, and the IP_3_ binding domain of the IP_3_ receptor type 1, 2, or 3 and can be targeted to the cytosolic or nuclear compartment. An increase in [IP_3_] is detected by an increase in FRET signals and a change in the YFP/CFP fluorescence ratio.

Further progress in the study of IP_3_-dependent Ca^2+^ signaling became possible with the development of caged IP_3_ compounds (Smith et al., 2009). Upon UV-light dependent photolysis, IP_3_ is released in its biological active form and can be readily used to study this signaling pathway without possible additional effects of GPCR stimulation other than IP_3_ generation (i.e., effects mediated by diacylglycerol that is generated concomitantly with IP_3_ by PLC). These approaches can be used in parallel, as shown in Figure 2A: a cardiomyocyte expressing FIRE-1-cyt exhibits an increase in the FRET signal of ~6% upon IP_3_ uncaging, indicative of a detectable change of global cytosolic [IP_3_]. Moreover Figure 2 depicts the influence of IP_3_ uncaging on different cellular compartments in atrial and ventricular cells. Figure 2B exemplifies the small impact of IP_3_ uncaging on local cytosolic and nuclear Ca^2+^ transients in field stimulated (0.5 Hz) ventricular cells (Fluo-4). Only prolonged exposure to the IP_3_ uncaging signal (100 ms laser illumination) has immediate visible effects on local Ca^2+^ release (Figure 2C). The IP_3_ effects on diastolic [Ca^2+^]_i_ and the Ca^2+^ transient amplitude are particularly pronounced for the nuclear region. As compared to ventricular cells, atrial myocytes are more sensitive to IP_3_ uncaging at smaller laser exposure durations (2 ms; i.e., smaller [IP_3_]) and the overall effect on cytosolic, nuclear and subsarcolemmal Ca^2+^ transient amplitudes is higher upon IP_3_ uncaging. Note also the altered Ca^2+^ transient kinetics with a prolongation of the Ca^2+^ transient's amplitude following IP_3_ uncaging (Figure 2C***a***). Figure 2C***b*** shows the effect of the IP_3_R blocker 2-APB (10 μM). The effect of IP_3_ uncaging on Ca^2+^ transients in an atrial cell, pre-incubated with 2-APB, was abolished.

## Mathematical approaches for simulating cytosolic and nuclear Ca^2+^ and IP_3_ signals

Computational modeling has proven to be a powerful approach to study cardiac physiology and its implications for disease. With increasing availability of biophysical and physiological data, mathematical models have also become more sophisticated. They provided new insights into how cellular structures, channels and receptor distributions or Ca^2+^/IP_3_ signaling regulate cardiac ECC. A number of *deterministic models* of ventricular and atrial myocyte electrophysiology, intracellular Ca^2+^ handling and bioenergetics have been published. For a more complete review on successes and failures in these modeling pursuits we refer the reader to some excellent recently published articles (Noble, 2011; Jafri, 2012; Noble et al., 2012; Sobie and Lederer, 2012; Poláková and Sobie, 2013; Wilhelms et al., 2013). Several *computational models* have been constructed to investigate IP_3_ synthesis and the sub-cellular mechanisms regulating IP_3_R-mediated Ca^2+^ signaling. The first model of an IP_3_ signaling system, built to simulate IP_3_ signals in response to stimulation with cardiac hypertrophic neurohumoral agonists like Endothelin-1 and Angiotensin II, was published by Cooling et al. (2007). The key controlling parameters with respect to the resultant cytosolic [IP_3_] in atrial cells were identified, including phosphorylation of membrane receptors, ligand strength, binding kinetics to pre-coupled (with GαGDP) receptors and kinetics associated with pre-coupling the receptors. In 1992, De Young and Keizer (1992) constructed the first simplified model of the IP_3_ receptor. Subsequent theoretical studies, based on new experimental data, have investigated the complex dynamic properties of type 1, 2, or 3 IP_3_Rs (Li and Rinzel, 1994; Laurent and Claret, 1997; LeBeau et al., 1999; Moraru et al., 1999; Mak et al., 2001; Sneyd and Dufour, 2002; Dawson et al., 2003; Siekmann et al., 2012). Based on quantitative measurements of IP_3_R properties, several stochastic models of the single channel and channel-clusters have been constructed (Swillens et al., 1998; Shuai and Jung, 2002; Falcke, 2003; Fraiman and Dawson, 2004; Thul and Falcke, 2004; Gin et al., 2009). Fraiman and Dawson (2004) were the first to include an explicit dependence of IP_3_R gating on SR-luminal Ca^2+^. To investigate the mechanisms underlying pacemaker cell activity, Youm et al. (2006) developed a deterministic model that includes ion channels, NCX, pumps, the intracellular machinery for Ca^2+^ regulation, cytosolic IP_3_ production and IP_3_-mediated Ca^2+^ release activity. Their model supports the idea that the cyclic changes in cytosolic Ca^2+^ and IP_3_ play a key role in the generation of regenerative pacemaker potentials. *Spatiotemporal continuum models*, seeking to investigate the mechanisms of IP_3_-mediated Ca^2+^ signaling in cells where IP_3_Rs are known to be the dominant Ca^2+^ release channels, have been published as well. Jafri and Keizer, combining a realistic model of IP_3_-induced Ca^2+^ oscillations with the diffusion of IP_3_ and buffered diffusion of Ca^2+^, developed a reaction-diffusion continuum model in Xenopus oocytes (Jafri and Keizer, 1994, 1995). Their results suggest that Ca^2+^ diffusion, which was much slower than that of IP_3_ because of endogenous Ca^2+^ buffers, had only a small effect on predicted Ca^2+^ transients. These findings imply a possible previous undisclosed role for IP_3_ in cell signaling. Means et al. (2006) used a reaction-diffusion model to simulate Ca^2+^ and IP_3_ dynamics in mast cells. The model was built upon a 3D reconstruction of the endoplasmic reticulum (ER) geometry from electron-tomography series. This model simultaneously tracks the changes in cytoplasmic and ER [Ca^2+^], includes luminal and cytoplasmic Ca^2+^ buffers, plasma membrane Ca^2+^ fluxes, SERCA, ER leakage, and type-2 IP_3_R. A unique feature of the model is the inclusion of the stochastic behavior of type-2 IP_3_R. The results showed that IP_3_Rs in close proximity modulate the activity of their neighbors through local Ca^2+^ feedback effects. Finally, in 1999 an analysis performed by fluorescence measurements of [Ca^2+^]_i_ and [Ca^2+^]_nuc_ in ventricular myocytes revealed that [Ca^2+^]_nuc_ increases concomitantly with [Ca^2+^]_i_ upon electrical stimulation, but the pattern of [Ca^2+^]_nuc_ increase was biphasic (rapid and slow) (Genka et al., 1999). Both sets of [Ca^2+^]_i_ and [Ca^2+^]_nuc_ data were well fitted by predictions derived from a simplified model of Ca^2+^ diffusion across the NPCs with two different Ca^2+^ diffusion constants. A plausible explanation of this finding is that the change in [Ca^2+^]_nuc_ is caused by Ca^2+^ diffusion from the cytosol to the nucleus through NPCs, but the permeability of the NPCs shifts from free to moderately restricted during contraction (Genka et al., 1999). The partial restriction of Ca^2+^ diffusion into the nucleus at high [Ca^2+^]_i_ may support the idea of a defense mechanism protecting the nucleus against Ca^2+^ overload during cell contraction.

**Figure 3:**
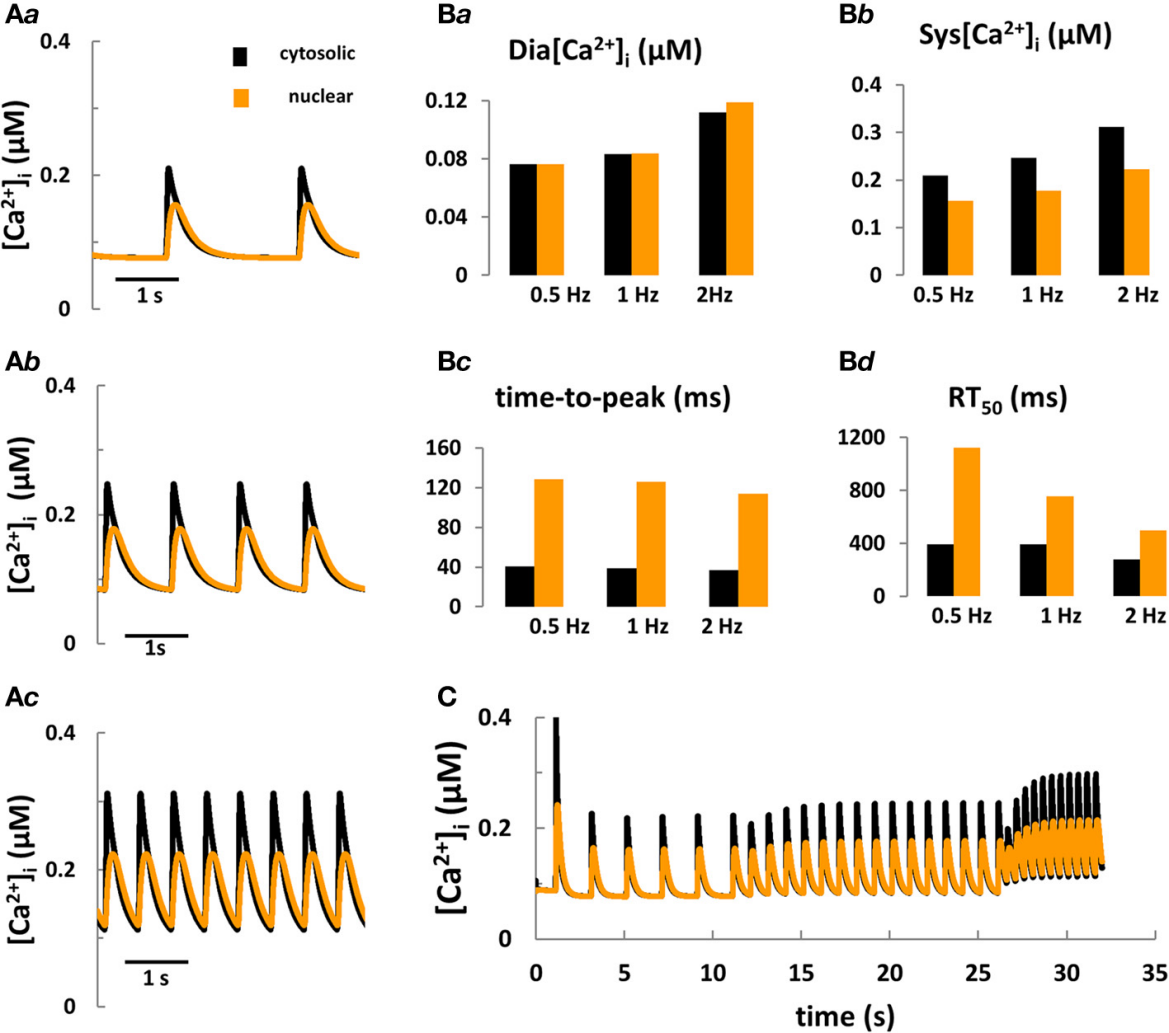
**Computer modeling of cytosolic and nuclear Ca^2+^ signals under control conditions.** (**A*a***–**A*c***) Predicted cytosolic and nuclear steady-state Ca^2+^ transients under control conditions in rabbit ventricular myocytes stimulated for 10 min at frequencies of 0.5, 1, and 2 Hz, respectively. **(B*a–*B*d*)** Predicted kinetic parameters of [Ca^2+^]_i_ and [Ca^2+^]_nuc_ transients: Dia [Ca^2+^], diastolic Ca^2+^; Sys [Ca^2+^], systolic Ca^2+^; RT_50_, time to 50% [Ca^2+^] relaxation. **(C)** Frequency-dependent changes in [Ca^2+^] in the nucleus vs. the cytoplasm during stepwise increases of the stimulation frequency from 0.5 to 2 Hz.

Taken together, the aforementioned modeling efforts fill a number of specific gaps of knowledge with respect to cell electrophysiology and cytosolic Ca^2+^ and IP_3_ signaling. To date, however, no quantitative model coupling the cell electrophysiology with Ca^2+^ and IP_3_ signaling in the cytosol and nucleus in cardiomyocytes exists. The development of a new system model, coupling ECC and ETC is important because: (a) this tool would provide fundamental new information on the role of IP_3_R-mediated Ca^2+^ signaling during ECC for arrhythmogenesis, for electrophysiological changes and for nuclear Ca^2+^ signaling in normal and failing cardiac cells; (b) as more experimental details on the complexity of IP_3_ regulation in myocytes accumulates, the intuitive interpretation of new findings becomes increasingly impractical and sometimes controversial. In pursuing this goal we extended the Shannon-Bers model in rabbit ventricular myocytes (Shannon et al., 2004). New equations, describing nuclear Ca^2+^ dynamics and its dependence on [Ca]_i_, nuclear Ca^2+^ buffering and transport via NPCs and NE (i.e., SR) were incorporated (see Figure 1; Michailova et al. unpublished data). Preliminary results (Figures 3A,B) show that the model predictions are in qualitative agreement with our Ca^2+^ transient measurements at 0.5 Hz electrical stimulation (see Figure 2B) and published experimental data (Ljubojevic et al., 2011) of global cytosolic and nuclear Ca^2+^ transients under control conditions, i.e., in absence of activation of IP_3_ signaling. The predicted [Ca^2+^]_i_ and [Ca^2+^]_nuc_ transients (and action potentials and [Ca^2+^]_SR_; not shown) are stable during 10 min stimulation at 0.5, 1, or 2 Hz. The model mimics also the frequency-dependent increases in the diastolic [Ca^2+^]_i_ (Shannon et al., 2004), but no obvious differences in diastolic levels of [Ca^2+^]_nuc_ vs. [Ca^2+^]_i_ at any given frequency were predicted. At each frequency the systolic Ca^2+^ peaks were lower in the nuclei and positive force-frequency increases in systolic [Ca^2+^]_i_ and [Ca^2+^]_nuc_ were predicted. The kinetic parameters of Ca^2+^ transients (time to peak and time to 50% [Ca^2+^] relaxation; RT_50_) were slower in the nucleus as compared to the cytosol. The physiological utility of the model was tested further by applying different frequencies to simulate the positive force-frequency relationship (Figure 3C). In agreement with experiments (Ljubojevic et al., 2011), upon increasing the rate from 0.5 to 2 Hz diastolic [Ca^2+^] and systolic Ca^2+^ peaks in the nucleus and cytoplasm increased in magnitude and the predicted amplitude of the Ca^2+^ transients were smaller in the nucleus compared to the cytosol.

## Conclusions and future perspectives

In this review we discussed the current state of experimental and modeling approaches to investigate nuclear and cytosolic Ca^2+^ homeostasis, whereby we focused on IP_3_-dependent Ca^2+^ signaling in adult myocytes. We presented experimental data from ventricular and atrial cells, showing the effects of sudden increases in [IP_3_] on nuclear and cytosolic Ca^2+^ transients during field stimulation as well as different approaches to study IP_3_-mediated Ca^2+^ release (i.e., FIRE-1-cyt as a tool to quantify [IP_3_], IP_3_ uncaging to mimic physiological increases in [IP_3_] and 2-APB to block IP_3_R mediated Ca^2+^ release). Moreover we compared experimentally the influence of IP_3_ uncaging on different compartments (nucleoplasm, cytosol) and were able to show that ventricular cells need a stronger IP_3_ stimulus to elicit a nuclear response, whereas atrial cells display substantial increases in nuclear and cytosolic Ca^2+^ transient amplitude upon a weaker IP_3_ uncaging stimulus, consistent with their higher total expression of IP_3_Rs as compared to ventricle. The recent development of FRET-based probes used for the detection of [IP_3_] as well as approaches to alter nuclear and/or cytosolic [IP_3_] provide experimental tools for the study of IP_3_-dependent Ca^2+^ release and its importance in ECC and ETC.

We also presented our recent efforts of a first attempt to develop an electrophysiological and Ca^2+^ signaling model that integrates three different cellular subsystems (cytosol, SR, nucleus) and couples Ca^2+^ dynamics in the cytosol and nucleus. This new tool is under development and will undergo further testing in its prediction of experimental [Ca^2+^]_nuc_ and [Ca^2+^]_i_ data in rabbit ventricular cells. The proposed model will also be extended to investigate how the complex dynamics of type-2 IP_3_ receptors (Sneyd and Dufour, 2002; Siekmann et al., 2012), the stochastic behavior of IP_3_R channel (Fraiman and Dawson, 2004) and/or the stimulation of IP_3_ signal transduction pathway with neurohumoral agonists (Cooling et al., 2007) regulate ventricular ECC and ETC. Furthermore, the mechanisms underlying IP_3_-induced positive inotropy in cardiomyocytes continue to be controversial with numerous cellular targets being implicated in the response, including L-type Ca^2+^ channels, K^+^ channels, and Na^+^/Ca^2+^ exchange (Lauer et al., 1992; Watanabe and Endoh, 1999; Woo and Lee, 1999; Yang et al., 1999; He et al., 2000; James et al., 2001; Zhang et al., 2001; Puglisi et al., 2011; Signore et al., 2013). The current model can be extended to investigate these effects as well. This model also provides a good quantitative framework to integrate reactions for calmodulin (CaM), calcineurin (CaN), CaMKII, and CaM buffering in the nucleus and can be coupled to the previously described and validated ECC models of CaM-CaMKII-CaN in rabbit ventricular cells (Hund and Rudy, 2004; Grandi et al., 2007; Saucerman and Bers, 2008; Bers and Grandi, 2009; Kraeuter et al., 2010; Soltis and Saucerman, 2010). This will allow testing hypotheses on how the interactions between Ca^2+^, IP_3_, and CaMKII signaling pathways contribute to heart failure phenotypes. Finally, the tools and insights our group develops will be useful to investigate how perturbations in cytosolic and nuclear Ca^2+^ and IP_3_ signaling affect ECC and ETC in atrial myocytes (Grandi et al., 2011; Koivumäki et al., 2011).

## Author and contributors

Designed the work: Anushka P. Michailova and Felix Hohendanner. Performed the experiments: Felix Hohendanner and Lothar A. Blatter. Performed the simulations: Anushka P. Michailova. Analyzed the data: Anushka P. Michailova, Felix Hohendanner, Lothar A. Blatter, and Andrew D. McCulloch. Contributed reagents/materials/analysis tools: Anushka P. Michailova, Felix Hohendanner, and Lothar A. Blatter. Wrote the paper: Anushka P. Michailova, Felix Hohendanner, Lothar A. Blatter, and Andrew D. McCulloch.

### Conflict of interest statement

The authors declare that the research was conducted in the absence of any commercial or financial relationships that could be construed as a potential conflict of interest.
